# Interfacing With the Electronic Health Record (EHR): A Comparative Review of Modes of Documentation

**DOI:** 10.7759/cureus.26330

**Published:** 2022-06-25

**Authors:** John P Avendano, Daniel O Gallagher, Joseph D Hawes, Joseph Boyle, Laurie Glasser, Jomar Aryee, Brian M Katt

**Affiliations:** 1 Department of Orthopaedic Surgery, Rutgers Robert Wood Johnson Medical School, New Brunswick, USA; 2 Physical Medicine and Rehabilitation, Orthopaedic Institute Brielle Orthopaedics, Wall, USA

**Keywords:** standardized template, charting, artificial intelligence in healthcare, electronic health record (ehr), documentation errors, clinical documentation improvement, documentation workflow, digital scribe, scribe

## Abstract

Electronic health records (EHRs) have provided physicians with a systematic framework for collecting patient data, organizing notes from the healthcare team, and managing the daily workflow in the modern era of healthcare. Despite these advantages, EHRs have proven to be problematic for clinicians. The burdensome regulations requiring increased documentation with the EHR paradigm have led to inefficiencies from data-entry requirements forcing physicians to spend an inordinate amount of time on it, affecting the time available for direct patient care as well as leading to professional burnout. As a result, new modalities such as speech recognition, medical scribes, pre-made EHR templates, and digital scribes [a form of artificial intelligence (AI) based on ambient speech recognition] are increasingly being used to reduce charting time and increase the time available for patient care. The purpose of our review is to provide an up-to-date review of the literature on these modalities including their benefits and shortcomings, to help physicians and other medical professionals choose the best methods to document their patient-care encounters efficiently and effectively.

## Introduction and background

Documentation of the physician-patient interaction including the history and physical exam (H&P), assessment of medical problems, counseling, and description of services rendered is a necessary but often vexing task in healthcare. While documentation is not necessarily required for effective delivery of care, without a record of the interaction, other clinicians may not have access to the recommendations of the treating healthcare provider. The value added by electronic health records (EHRs) includes accessibility and portability of patient-specific information. In theory, this should help prevent duplicative work by storing large volumes of information in a way that clinicians can efficiently access both locally and remotely.

Providers have been required to document increasingly greater volumes of data about their patient interactions because of fiscal, insurance-related, and medicolegal factors. As per recent studies, physicians spend up to 35% of their time on documentation [1]. Time spent on data entry into the EHR not only eats into the time available for direct patient care but also the clinician’s personal time. It has been estimated that for each hour spent on direct patient care, up to two hours are spent on EHR-related activities [2,3]. Further, as computerized data entry interfacing with the EHR has replaced conversations with patients, it is no surprise that the phenomenon of physicians reporting burnout is on the rise [4].

Several innovative ways to decrease the burden of data entry and interfacing with EHRs have been developed, including the appointment of additional personnel (scribes and office assistants), advancements in EHR interface software (templated documentation, smart keys), add-ons to EHR interface software (dictation services), and the use of artificial intelligence (AI) in digital scribing systems (digital scribes).

## Review

Speech recognition in electronic health records (EHRs)

The term “speech recognition systems” (SR) refers to systems that convert spoken words into written text. Simple versions of SR used in EHRs enable immediate voice-to-text transcription into the medical record, allowing for immediate proofreading. Natural language processing is used in more advanced speech recognition, using technology with voice-enabled ambient speech capability.

Several studies in the literature have investigated the efficacy and accuracy of SR (voice-to-text) transcription and have compared it to traditional typed data-entry patient charts. Dela Cruz et al. conducted a prospective observational study at two academic teaching hospitals’ emergency departments (EDs), one of which used traditional voice recognition software while the other used traditional typed data entry [5]. Observers accompanied attending physicians for 180-minute time frames, logging physician tasks at 30-second intervals [5]. There were no statistically significant differences in the amount of time physicians spent charting or the amount of time physicians spent on direct patient care [5]. Further, significantly more interruptions per hour occurred with provider-transcribed data entry when compared to that of speech recognition data entry [5].

Hodgson et al. also performed a study comparing SR to traditional typing, in which 35 ED clinicians were randomly assigned clinical documentation tasks to complete by using SR or a traditional keyboard and mouse [6]. The data collection process began in 2015 with the use of SR software Nuance Dragon Medical 360 Network Edition UK (version 2.0, released in 2013, Nuance Healthcare, Burlington, MA) [6]. The number of observed errors and completion times associated with each task were recorded [6]. When compared to a traditional keyboard and mouse, SR was found to be slower for both simple and complex tasks, and it increased the risk of documentation errors, including errors with the potential to cause clinical harm [6]. Numerous errors were attributed to factors relating to the configuration and integration of the SR system [6]. Due to the influence this may have had on the SR performance, the same group identified potential factors, revised the EHR and SR systems, and conducted a replication study in 2016 using Nuance Dragon Medical 360 Network Edition UK (version 2.4, released in 2015, Nuance Healthcare) [7]. Again, the results indicated that SR resulted in slower documentation times and an increase in the number of data-entry errors, including those with the potential to cause clinical harm [7]. This highlights the importance of careful chart review by the clinicians following the use of SR, further increasing the time required to complete charting tasks. As technology advances and newer program updates are released, the accuracy of SR software will continue to be an area requiring additional dedicated research.

Blackley et al.'s systematic review investigating the use of speech recognition for charting reported a quicker turnaround time for SR compared to standard transcription, but overall SR documentation time was not conclusively faster [8]. In a separate controlled observational study, Blackley et al. investigated dictated notes compared to typed notes, finding similarities in documentation time between the two cohorts, but demonstrating that dictated notes were longer than typed notes (320.6 vs. 180.8 words; p=0.004) on average with more unique words (170.9 vs. 120.4; p=0.001) [9]. The same study noted that typed notes had a larger number of uncorrected errors when compared to dictation-based notes (2.9 vs. 1.5), though most of which were minor misspellings [9]. Ultimately, it was concluded that dictated notes were better for creating comprehensive and more complete documentation [9].

Poder et al.’s systematic review compared SR to traditional typed documentation and found that speech recognition system notes showed lower recognition rates, higher error rates, and an increase in dictation times when compared to a transcriptionist [10]. Poder concluded that the main benefit of SR was the immediacy of the charted documentation [10]. Vogel et al. performed a randomized controlled trial where documentation by 28 physicians was randomized with or without speech recognition [11]. Physician mood was also assessed via a self-reporting assessment scale ranging from 1 to 3, with lower values indicating greater satisfaction [11]. Out of 1,455 reports, 718 were assisted by SR, and 737 were not [11]. The average number of characters per minute using SR was 217, which was greater than the 173 characters per minute without SR, but the study did not conduct an assessment of accuracy [11]. The authors concluded that medical documentation with the assistance of speech recognition leads to improvements in documentation speed, document length, and participant mood when compared to self-typing [11].

There is no clear consensus on the benefits of speech recognition over transcription/self-typing for charting purposes, though there is the potential for its use in certain clinical situations like the ones encountered in EDs, which require longer and more descriptive documentation. Variables such as turnaround time, accuracy, documentation time, and recognition rates were measured but the results are inconclusive, mainly because there was low subject participation and poor stratification of providers. Further studies will be needed to assess the ways in which speech recognition can increase the accuracy and decrease the documentation time, without creating more issues from impaired speech recognition.

Medical scribes

Medical scribes have grown in popularity as a time management tool for many providers and healthcare systems. Scribes are individuals who type and organize the encounter notes on behalf of the clinician, frequently in real time. Their role is adaptable, as they can accompany the provider to each encounter, be stationed in a workspace nearby for dictation following the encounter, or be present remotely via internet connection. The estimated cost of a scribe is $50,000 a year plus training costs of $6,317 per scribe [12].

The rapid adoption of the services of medical scribes over the last several years has been attributed to their perceived efficiency and benefits in reducing the burden of documentation; however, this notion has been challenged by a variety of sources. For example, Heaton et al. concluded in a systematic review and meta-analysis of the use of medical scribes in the ED that there was no difference in length of stay or provider-to-disposition time between visits [13]. Although patients' stay in the hospital did not decrease, the authors reported that doctors using scribes saw a greater number of patients per hour (0.17 more per hour) and received higher reimbursements secondary to improved documentation [13]. The exact quantification of the potential financial benefit of scribes was challenging due to studies reporting a variety of financial metrics, such as total dollar amounts, incremental savings, and relative value units (RVUs) [13]. However, it is clear that due to the expenditures associated with educating and training a scribe, it is more economical to employ scribes who can work for a longer period of time [12,13].

Hasan et al. conducted a study in which consultation time at a level 1 trauma center was examined, comparing 151 patients seen with a scribe and 304 without a scribe [14]. The mean consultation time was 3.2 minutes shorter in the scribe group compared with the control group of physicians responsible for completing their notes independently [14]. Gottlieb et al. also concluded that the addition of scribes improved RVUs per hour, RVUs per encounter, and the number of patients seen per hour [15]. When working with a scribe, providers were able to bill a higher level of evaluation and management code. However, scribes were not found to improve ED length of stay despite the intended purpose of improving the efficiency of physician encounters [15].

Hasan et al. evaluated physician satisfaction in the setting of medical scribe use [14]. Physicians reported increased satisfaction with medical scribing services, greater overall productivity (patients seen per hour), and cost savings due to reduced time spent charting per patient [14]. Tohmasi et al. in an electronic survey study on general surgery residents and faculty using outpatient scribes concluded that the implementation of medical scribes enhanced the residents’ education and wellness by mitigating resident fatigue, improving adherence to duty hour restrictions, and allowing faculty and residents more time to focus on patient care and education [16]. Similarly, Gottlieb et al. reported that the presence of a medical scribe improved both provider and patient satisfaction [15]. Keefe et al. concluded that the presence of a medical scribe had no effect on patient satisfaction based on a retrospective survey assessing wait times and provider satisfaction [17].

EHRs are less ideal in practice than they are in theory due to their unwieldy user interfaces and time-consuming nature for physicians' day-to-day workflow. As such, medical scribes can be a valuable asset to physicians, assisting them in maximizing reimbursement, productivity, and physician satisfaction. However, it is unknown whether EHR implementation in the clinical setting will result in a reduction in patient length of stay because of the intended purpose of documentation improvements leading to more efficiency, which would result in faster throughput.

EHR shortcuts and templates

Templated formats have become more prevalent as a means of easing the burden of physician documentation. Numerous EHR software programs are now available, including a comprehensive library of pre-formatted documentation outlines customized for specific injuries or illnesses. Cao et al. compared EHR templated forms to non-templated formats in documenting comprehensive neurologic and vascular examinations, with a focus on early neurovascular injury detection [18]. The authors reported a significant improvement in the thoroughness in the documentation of vascular, motor, and sensory examinations in the template group, and noted that neurological injuries were more likely to be identified in the template group compared to the non-template group [18]. They concluded that templates increased the accuracy and timeliness of documentation, as well as the clinician's ability to detect preoperative neurological injuries [18].

Urchek et al. reported that when residents used an orthopedic template to document the H&P of 42 pediatric supracondylar humerus fractures, the results were more complete compared to non-templated notes while maintaining comparable levels of accuracy [19]. Copley et al.’s survey among the members of the Pediatric Orthopaedic Society of North America regarding their use and satisfaction with electronic medical records (EMRs) reported that 41.9% found templates for specific conditions helpful and 59.8% wanted improved templates for specific conditions [20].

The use of templated documents was shown to improve preoperative diagnosis, documentation accuracy, documentation time to completion, and physician satisfaction.

Digital scribes (artificial intelligence)

The future of medical documentation may undergo dramatic changes as a result of AI's rapid advancement and the numerous applications it may create in healthcare. Digital scribes or computer-assisted documentation are an emerging medical application of AI to automate clinical documentation. A microphone is used to record physician-patient conversations and transcribe them to text using automatic speech recognition software. Natural language processing models then extract and summarize relevant information to the physician, which can be used to complete clinical notes, add billing codes, or support a diagnosis with specific extracted information [21].

In clinical practice, a digital scribe not only has the ability to compose documentation using voice-to-text software but can also assist physicians with AI-based diagnostic and treatment decisions. The purpose of employing scribes is to decrease physician time spent behind a computer typing medical notes, allowing for increased patient-physician interaction. While EHRs in their current form may improve the quality of clinical documentation, they frequently result in increased documentation times, decreased clinician satisfaction, decreased length of patient interaction, new sources of risk to patient safety, and substantial investment costs for providers [4,22,23]. Consequently, as medicine evolves, addressing these various factors via improved methods of charting is critical.

Given the many frustrations clinicians have expressed with the current EHR model, some envision the future of charting materializing as a hybrid of human and computer-driven documentation. Given the difficulties providers often face when integrating speech recognition with proofing into EHRs, mixed-initiative documentation systems hold significant promise due to their ability to solve many difficult problems seen in natural language processing. The ability to construct individualized interactions between humans and computers can optimize and improve EHR functionalities [24]. Further, “autopilot” digital scribes (i.e., automated computer AI) could lead a context-aware, adaptive documentation process, where human interaction would then only be required to assist the machine, if necessary. To assess the utility of digital scribes, Wang et al. conducted a simulated patient encounter study in which one medical student acted as the provider and was responsible for editing the note, and another student acted as a simulated patient [25]. The digital scribe was 2.7 times faster than typing and dictating for H&P sections [25]. This study showed that the benefits of utilizing this form of AI documentation pertain to providers requiring minimal training, and suggests that providers would have significant improvement in their efficiency within two days of use [25]. This study was limited by the simulated nature of the patient encounters and a lack of a control group to assess relative levels of student experience in typing vs. dictating [25].

Regardless of their perceived benefits, Coiera et al.'s study has raised concerns about the level of power that digital scribes would cede to technology, with a risk of potential patient harm caused by any inaccuracies [24]. There are also potential medicolegal ramifications regarding the transition from the record being produced by humans to a machine-generated summary. Digital scribes also have the potential to ruin the sanctity of the clinician-patient relationship, though this ultimately will come down to the way in which virtual interactions are designed with scribes. Quiroz et al. further elaborate on some of the challenges one might see with the development of an automated speech-based documentation system in clinical settings [26]. The authors highlight current technological limitations such as minimizing ambient noise, extracting medical concepts, differentiating multiple speakers (patient vs. family member vs. physician), and organizing unstructured conversations [26]. Additionally, the authors note that physicians frequently combine clinical and documentation responsibilities throughout the day, allowing for time to reflect on each case and structure their decision-making with input from each patient encounter that provides useful context for the next [26]. Therefore, there is a concern that the symbiotic relationship between patient encounters and documentation may be lost with the advent of digital scribe technology [26].

When considering AI and the concept of a "digital scribe," it is necessary to keep in mind that the field of AI remains largely unexplored and is still constrained by current technological system processing. While the available literature suggests the promise of a future in which technology is better able to alleviate many of the burdens we currently face, we must keep in mind that broad-based shifts in modalities always bring about their own set of new challenges. As such, we should remain cautiously optimistic about the potential of human- and EHR-based interactions. While it appears that there is great potential for future collaborations between clinicians, patients, and technology, the onus is on us to be visionaries in re-imagining the future of EHR documentation [24].

The numerous modalities of documentation described in the above paragraphs, along with their respective benefits and drawbacks, are summarized in Table 1.

**Table 1:** A summary of the benefits and drawbacks of the various modalities discussed

Modality	Potential benefits	Drawbacks
Voice recognition/dictation	More detailed documentation [9]. Decreased turnaround time between patient encounters [9]. Fewer grammatical errors vs. typed notes [9]	Conflicting evidence for improvements in turnaround times and error rates vs. traditional typed notes [5,6]. Unclear consensus [8]
Medical scribes	Improvement in physician and resident wellness [14,16]. Decreased physician burnout[13,14]. Increased turnaround time [13]. Increased relative value units (RVUs) and billing[13-15]	No improvement in patient satisfaction[15,17]. Unchanged patient length of stay [13]. Unchanged time taken to ultimately discharge patient [15]
Preset templates	Higher likelihood of completing and documenting more detailed physical examination findings[17,18]. Increased detection of concomitant injuries [18]. More efficient chart completion[18]	Unchanged accuracy of clinical documentation[17]. May not correlate with the specific clinical picture, requiring burdensome clinician edits [20]. Relatively low physician satisfaction[20]
Artificial intelligence (AI)	Providing a synergistic relationship between software and clinician [11]. Faster completion of history and physical examination sections vs. typing or dictation [11]. Theoretical machine learning capability to increase accuracy and speed over time [24]	Ethical concerns regarding machine-generated documentation[23]. Few examples of real-world implementation [24]

## Conclusions

Medical documentation has evolved into a significant responsibility for the modern physician. As a result, various mechanisms to improve its integration into clinical practice with minimal disruption have been investigated; however, the current literature has a dearth of evidence-based studies to support any one initiative over another. Speech recognition for charting purposes has been reported to provide more comprehensive documentation and fewer interruptions per hour. When compared to controls in the various studies investigated, medical scribes and custom-built templates improved patient satisfaction, overall quality of the documentation, and overall efficiency in terms of patients seen per hour. AI is an exciting emerging field, but studies have not yet been able to quantify its utility. Future research is needed to investigate the synergistic use of multiple modalities, as each has a unique set of attributes that improve certain aspects of documentation.

Though there will never be a perfect or "one size fits all" method for providers to complete patient charts, it is important to keep track of various preferences to determine trends among clinicians’ needs. It is our hope that our review will serve to increase understanding regarding various options available for EHR documentation.
